# Incidental lesion in oncology patients: kidneys

**DOI:** 10.1186/1470-7330-14-S1-O44

**Published:** 2014-10-09

**Authors:** Aslam Sohaib

**Affiliations:** 1 Dept. of Radiology, Royal Marsden Hospital, London & Sutton, UK

## 

The increasing use of cross-sectional imaging has led to greater detection of incidental lesions in oncology and non-oncology patients. These incidental lesions are unexpected and usually asymptomatic abnormalities that are identified when imaging for other purposes. These incidental lesions create a diagnostic and management challenge for radiologists and clinicians [1]. They may result in patients having unnecessary investigations and treatment, which is not without risks or expense. The American College of Radiologists has developed guidelines and recommendations for incidentally detected lesions on abdominal imaging [2]. The guidance developed addresses incidental findings in the kidney, liver, adrenal glands and pancreas but does not deal specifically with cancer patients.

In the case of an incidentally detected renal lesion in a cancer patient, the incidental abnormality may represent metastatic disease, a second primary malignancy or a benign lesion. The diagnosis and management of such incidental findings will depend in part on the clinical setting, the pathology and stage of underlying primary malignancy and the imaging features of the incidental abnormality [3-6].

In terms of imaging characterisation many incidental kidney lesions can be fully characterised using ultrasound, CT or MRI. In characterising a renal mass, in general it is important to first ensure that the mass is not the result of non-neoplastic conditions that may mimic a tumour. For example focal bacterial pyelonephritis, pseudotumours such as columns of Bertin, hypertrophied tissue adjacent to scars, vascular anomalies, aneurysms and infarcts.

Further imaging characterisation may suggest that the lesion is a benign cystic renal tumour, i.e. Bosniak I or II lesions. If the lesion is solid, unless there are features in keeping with an angiomyolipoma (AML), it is usually not possible to differentiate benign from malignant primary tumour or metastases to the kidney. Therefore either cystic lesions or solid lesions in which malignancy cannot be excluded may need further assessment and this is an indication for a renal biopsy.

Metastases to the kidney are very uncommon. Renal metastases frequently do not present clinical symptoms and many patients have no haematuria. Traditionally, metastases to the kidney are thought to be multiple and bilateral, however descriptions are limited to case reports. The most common malignancies metastasing to the kidney are lung and breast cancer. Previous imaging or history is also useful in evaluating metastases to the kidney as there is often widespread metastatic disease with renal involvement. Isolated metastasis is very rare and may need a biopsy in order to distinguish it from primary renal neoplasm.
